# 3D quantitative MRI for cognitive performance: T1 relaxation times of the right putamen and volumes of the left hippocampus as key biomarkers

**DOI:** 10.1016/j.ynirp.2026.100351

**Published:** 2026-05-07

**Authors:** Lora Kovacheva, Jan R. Schüre, Svenja Klinsing, Rafael Willems, Mario Balo, Ralf Deichmann, Elke Hattingen, Christophe T. Arendt

**Affiliations:** a Goethe University Frankfurt, University Hospital, Institute of Neuroradiology, Frankfurt, Germany; b Goethe University Frankfurt, Cooperative Brain Imaging Center - CoBIC, Frankfurt, Germany; c Goethe University Frankfurt, University Hospital, Department of Neurology, Frankfurt, Germany

**Keywords:** Cognitive dysfunction, qT1, MRI, Dementia, Putamen

## Abstract

**Background:**

Mild cognitive impairment (MCI) represents anearly stage of cognitive decline, often preceding dementia. While hippocampal atrophy is a recognized imaging marker, the role of deep gray matter structures and tissue alterations remains less understood. This study aimed to assess whether quantitative T1 (qT1) relaxometry and volumetric measures derived from synthetic MRI are associated with global cognitive performance, as measured by the Montreal Cognitive Assessment (MoCA).

**Methods:**

In this cross-sectional study, 74 healthy adults (mean age 43.7 years, 50% female) underwent 3D-synthetic MRI and MoCA testing. Participants were stratified into cognitively normal (MoCA ≥26) and MCI (MoCA <26) groups. Brain volumes and qT1 values were extracted across regions. Associations with MoCA were assessed using multivariate regression, controlling for demographic and clinical covariates.

**Results:**

Left hippocampal volume was significantly reduced in the MCI group (p = 0.019) and was positively associated with MoCA scores (R^2^ = 0.18). Although qT1 values did not differ significantly between groups, qT1 in the right putamen independently predicted MoCA scores when adjusted for age (combined model R^2^ = 0.22). Age was also associated with cortical gray matter qT1 (r = −0.33, p = 0.005) and increased CSF volume (r = 0.45, p < 0.0001), indicating age-related structural changes. No significant effects of sex, BMI, vascular risk, or comorbidities were observed.

**Conclusions:**

Hippocampal volume and putamen qT1 are independent imaging correlates of cognitive performance. qT1 mapping may detect early subtle tissue changes not captured by conventional volumetry, supporting its potential role as a biomarker in cognitive aging.

## 1 Introduction

Mild cognitive impairment (MCI) is a common condition that affects 10–20% of adults over the age of 65 and is often considered a transitional phase preceding dementia (Langa and Levine, 2014; Gauthier et al., 2006). Early detection of MCI can potentially help slow down or prevent the progression of cognitive decline, as it is often considered a precursor to dementia. The Montreal Cognitive Assessment (MoCA) is a widely used screening tool developed in 2005 to detect MCI, offering high sensitivity and reduced bias compared to the Mini-Mental State Examination (MMSE) (Nasreddine et al., 2005; Folstein et al., 1975). MoCA evaluates multiple cognitive domains, including attention, memory, language, and visuospatial skills, with scores below 26 indicating potential impairment (Nasreddine et al., 2005).

Despite its clinical utility, diagnosing MCI remains challenging due to the absence of standardized diagnostic criteria and the heterogeneity of underlying pathologies (Langa and Levine, 2014; Gauthier et al., 2006). Both neurodegenerative and cerebrovascular mechanisms are implicated, with hallmark features such as beta-amyloid plaques and tau tangles contributing to neuronal dysfunction and atrophy (Gauthier et al., 2006; Mufson et al., 2012; Small et al., 2006). Small vessel disease and ischemic lesions may further exacerbate cognitive decline by impairing neuronal integrity and oxygenation (Gauthier et al., 2006).

While hippocampal atrophy is a well-established imaging marker in MCI and early Alzheimer's disease (AD), increasing evidence highlights the relevance of deep gray matter (GM) structures—including the thalamus and basal ganglia—as early sites of pathological change (Tuokkola et al., 2019; Van De et al., 2021; Dicks et al., 2018). These regions are crucial for cognitive-motor integration and are vulnerable to both neurodegeneration and vascular insults (Tuokkola et al., 2019; Van De et al., 2021; Dicks et al., 2018). Meta-analyses and voxel-based morphometry studies have shown volumetric loss in subcortical nuclei in MCI, sometimes preceding hippocampal changes (Dicks et al., 2018). Tuokkola et al. demonstrated that individuals with MCI exhibited reduced volumes in the right putamen compared to healthy controls, reinforcing its role in early disease stages (Tuokkola et al., 2019).

Magnetic resonance imaging (MRI) also enables quantitative assessment of tissue integrity through T1 relaxometry. The T1 relaxation time (qT1) represents an apparent relaxation parameter influenced by multiple hydrogen pools and therefore reflects tissue composition and alterations, including variations in water content (Fatouros et al., 1991; Neeb et al., 2006), (de)myelination (Jonkman et al., 2015; Steen et al., 1997; Eminian et al., 2018), iron accumulation (Stüber et al., 2014; Gelman et al., 2001), and gliosis (Jonkman et al., 2015). Recent studies using T1-mapping have shown widespread age-related differences in cortical tissue properties across the adult lifespan, suggesting alterations in myelin, iron, and water content with aging (Weiskopf et al., 2021; Callaghan et al., 2014). In line with this, surface-based qMRI analyses have reported age-related reductions in cortical T1 values across adulthood, particularly in frontal and temporal regions (Seiler et al., 2020). Age-related increases in qT1 have been also reported in deep GM structures such as the putamen, thalamus, and hippocampus, with accelerated changes observed after the age of 50 ^20^.These findings suggest that qT1 may be a sensitive biomarker of early neurodegenerative processes.

In this study, we examine whether qT1 metrics in deep GM—particularly the putamen—are associated with cognitive performance in individuals stratified by MoCA scores. Our goal is to assess the utility of qT1 as a potential imaging biomarker for MoCA-defined MCI.

## 2 Methods

### 2.1 Selection of participant population

This retrospective observational study was approved by the local ethics committee and was conducted in accordance with the Declaration of Helsinki and other applicable guidelines governing research involving human participants. Adult participants who voluntarily provided written informed consent were included in a local research database comprising neuroimaging and cognitive data. The imaging data are maintained at the Brain Imaging Center of Goethe University Frankfurt in collaboration with the Institute of Neuroradiology at the University Hospital Frankfurt. Consecutive individuals who had both completed the MoCA test and undergone a standardized research brain MRI scan were identified from this database as part of ongoing research protocols. Exclusion criteria included standard contraindications to MRI, as well as potential confounding factors for the assessment of cognitive function or brain structure, namely, a history of prior or current neuroinflammatory or systemic inflammatory diseases, known neurological or neuropsychiatric disorders, or previous intracranial surgery.

### 2.2 Baseline clinical data and cognitive assessment

Prior to MRI, all participants completed a standardized questionnaire capturing demographic and clinical information, including age, sex, smoking status, alcohol consumption, body mass index, and pre-existing medical conditions. These conditions were systematically categorized into four domains based on their potential relevance to cognitive function and brain structure:

• Cardiovascular factors: arterial hypertension, diabetes mellitus, arrhythmia, hyperlipidemia, heart infarction, obstructive sleep apnea syndrome (OSAS);
• Neurological/Psychiatric conditions: bipolar disorder, depression, migraine, concussion, insomnia;
• Metabolic conditions: hypothyroidism, anemia, vitamin D deficiency, vitamin B12 deficiency, iron deficiency, polycystic ovary syndrome (PCOS);
• Autoimmune/Inflammatory conditions: rheumatoid arthritis, asthma, neurodermatitis, eczema, psoriasis, fibromyalgia.

Each diagnosed condition within a category contributed one point to a category-specific burden score. Other recorded conditions—such as disc herniation, urinary tract infections, alopecia, pneumothorax, sinusitis, duodenal ulcer, and rare hematologic disorders (e.g., Factor V deficiency)—were documented but excluded from categorization due to unlikely relevance for cognitive function or brain morphology.

Cognitive performance was assessed using version 8.1 of MoCA. It evaluates short-term memory, visuospatial skills, executive function, attention, language, and orientation via tasks like delayed recall, clock drawing, trail making, serial subtraction, object naming, sentence repetition, and orientation questions. Scores range up to 30, with ≥26 considered normal. Participants were stratified according to MoCA scores (≥26 vs. <26), which served as a screening-based categorization of cognitive performance. This grouping represents a screening-based classification of cognitive performance rather than a formal clinical diagnosis of mild cognitive impairment. Consequently, several individuals in this subgroup had only mildly reduced scores. One point is added for individuals with ≤12 years of formal education.

### 2.3 MRI datasets acquisition and generation of synthetic anatomical images

MRI datasets were acquired on a whole-body 3 T scanner (MAGNETOM Prisma, Siemens Healthineers, Erlangen, Germany) at our Brain Imaging Center. A bore-integrated body coil was used for radiofrequency (RF) transmission, while a head coil (20-channel phased array) was employed for signal reception. Table 1 provides detailed technical parameters of the MRI sequence protocol. Following a planning localizer, a two-dimensional T2-weighted fluid-attenuated inversion recovery (FLAIR) sequence with a slice thickness of 3 mm was acquired in the axial plane, incorporating fat saturation. It was acquired for visual assessment of structural abnormalities and white matter hyperintensities (*e.g.*lacunar infarcts) but was not included in the quantitative imaging analyses. Subsequently, two three-dimensional gradient echo sequences were obtained with isotropic voxel dimensions of 1 mm^3^. These sequences differed solely in their flip angles, set at 4° and 24°, respectively, implementing the variable flip angle method (Venkatesan et al., 1998; Preibisch and Deichmann, 2009a). This approach involves acquiring one proton density (PD)-weighted and one qT1-weighted sequence, enabling the quantification of qT1 relaxation times through the signal intensity differences between the two acquisitions. Both datasets were acquired using a FLASH-EPI hybrid readout covering the entire brain in the sagittal plane (Preibisch and Deichmann, 2009a). To correct these values for inhomogeneities in the static magnetic field (B0) and the radiofrequency transmit field (B1), additional mapping sequences were employed. In detail, B0 mapping used the standard approach of acquiring two gradient echo data sets with different echo times (TE) and B1 mapping was based on a concept described in the literature (Volz et al., 2010). Custom-developed software was utilized to compute B0- and B1-corrected qT1 maps, integrating tools from MATLAB (MathWorks, Natick, MA) and the FMRIB Software Library (FSL, v5.0, Oxford, UK) (Volz et al., 2010; Nöth et al., 2015; Gracien et al., 2019). The qT1 calculation comprised additional corrections for incomplete RF spoiling (Preibisch and Deichmann, 2009b). As part of the post-processing pipeline, synthetic anatomical images were generated from these qT1 maps, which are technically similar, but offer an improved contrast-to-noise ratio and a substantially enhanced signal uniformity as compared to the conventional T1-weighted magnetization-prepared rapid acquisition gradient echo (MPRAGE) sequence. Furthermore, this concept allowed to synthesize MPRAGE contrasts for different target parameter sets based on a single qT1 map. The calculation is based on deriving pseudo-PD maps from the qT1 maps (Volz et al., 2012) and integrating them into a quantitative signal modeling framework to simulate MPRAGE-like contrast (Nöth et al., 2015). The two flip-angle datasets used for qT1 mapping were co-registered and combined to minimize motion-related inconsistencies between acquisitions and to generate a single motion-consistent anatomical image. The resulting synthetic images underwent additional corrections, including spatial realignment to account for anatomical misregistrations occurring during image acquisition. These corrections enhanced the geometric fidelity of the synthetic volumes, thereby improving their suitability for subsequent tissue segmentation, volumetric measurements and morphometric analyses. Image quality was controlled during acquisition. If artifacts appeared, the image protocol was repeated. The full MRI protocol was acquired for all participants included in the final analysis. Datasets underwent visual quality check for further artifacts (e.g. residual motion, ghosting, ringing, or slice dropouts). To reduce contamination from non-parenchymal signal contributions and partial volume effects (e.g. cerebrospinal fluid or tissue abnormalities), an 80% threshold was applied to partial volume estimates during segmentation.

**Table 1:** Technical acquisition and synthetic reconstruction parameters of the study MRI protocol.

	FOV [mm^3^]	Voxel Size [mm]	TR/TE/TI [ms]	Flip Angle [°]	Bandwith [Hz/pixel]	Acquisition Time [min:sec]
T2-weighted 2D FLAIR sequence	210 x 210 x 149	0.8 x 0.8 x 3	9000/81/2500	150	283	4:14
3D GRE VFA for qT1 mapping	256 x 224 x 160	1 x 1 x 1	16.4/6.7/-	4 & 24	222	4:54 & 4:54 i.e. total 9:48
Mapping of the static magnet field (3D dual-echo B0)	256 x 224 x 160	4 x 4 x 4	560/4.89 & 7.35/-	60	200	1:03
Mapping of the radiofrequency transmit field (3D GRE B1)	256 x 224 x 160	4 x 4 x 4	11/5/-	11	260	1:45
3D-Synthetic MPRAGE sequence (a)	256 x 224 x 160	1 x 1 x 1	1900/0/900	9	-	-

FLAIR fluid-attenuated inversion recovery, FOV field of view, MPRAGE magnetization-prepared rapid acquisition gradient-echo, TE echo time, TI inversion time, TR repetition time.

a The parameters shown for Synthetic MPRAGE are not actual acquisition parameters but rather the target parameters of an MPRAGE sequence for which contrasts were synthetically generated.

### 2.4 MRI data analyses

The synthetic MPRAGE datasets were subjected to fully automated anatomical segmentation using customized processing scripts based on FreeSurfer software suite (version 6, Martinos Center for Biomedical Imaging, Massachusetts General Hospital, Boston, MA). Specifically, it included the delineation of cerebrospinal fluid, as well as hemisphere-specific (left and right) global white matter and cortical gray matter, in addition to the brainstem and fourth ventricle. Key subcortical GM structures segmented as part of this process included the thalamus, caudate nucleus, putamen, globus pallidus, amygdala, hippocampus, and nucleus accumbens. To ensure accuracy of the anatomical parcellations, all segmentations were visualized and manually inspected using ITK-SNAP (version 3.6.0), an open-source software tool for medical image annotation. This platform was also employed to extract qT1 values and voxel counts for each defined region of interest. Tissue volumes were subsequently calculated by multiplying the number of voxels within each region by the voxel resolution of 1 mm^3^, yielding absolute volumetric measurements in cubic millimeters.

### 2.5 Statistical analyses

Statistical analyses were conducted using Python scripts (Python 3.11, packages including pandas, scipy, statsmodels, matplotlib, and seaborn). The significance level was set at α = 0.050 and p-values <0.05 were considered statistically significant. The distribution of continuous variables was visually assessed using histograms. Variables were summarized as mean ± standard deviation or median with interquartile range [Q1–Q3], depending on their distribution. For comparisons between individuals with and without MCI, unpaired two-tailed t-tests were used for normally distributed variables. To account for multiple comparisons, both a Bonferroni correction and the two-stage step-up method by Benjamini, Krieger, and Yekutieli (FDR 10%) were applied. Associations between imaging parameters and MoCA performance were assessed using Pearson's correlation coefficient. To identify independent predictors of MoCA performance, multiple linear regression models with stepwise backward elimination were performed. Assumptions of linear regression, including normality of residuals and absence of multicollinearity, were verified using Q-Q plots and variance inflation factors.

## 3 Results

### 3.1 Participant demographics, clinical characteristics, and cognitive assessment

Seventy-four participants (37 females, 37 males; mean age 43.7 years, range 20–70) underwent MoCA testing and brain MRI. Based on MoCA scores, participants were classified as cognitively normal (MoCA ≥26, n = 61) or mildly impaired (MoCA <26, n = 13). Despite cognitive differences, no significant group differences were found in age, sex, BMI, smoking, alcohol use, or clinical variables such as Fazekas score, lesion load, infarctions, or NIHSS score (Table 2). The prevalence of cardiovascular, neuropsychiatric, metabolic, and autoimmune conditions was also similar across groups. These findings suggest that the observed cognitive differences were not attributable to major demographic, lifestyle, or clinical confounders.

**Table 2:** Demographic, clinical, and risk factor description of study groups.

		MoCA ≥26	MoCA <26	p-values
Number		61	13	-
MoCA-Score (Mean ± SD)		28.2 ± 1.4	23.1 ± 2.2	-
Age (y, Mean ± SD)		42 ± 13	48.7 ± 13.5	0.159
Sex (f: m) ∗		30 : 31	7 : 6	0.479
Weight (kg, Mean ± SD)		78.5 ± 15.2	78.9 ± 14.6	0.955
Height (m, Mean ± SD)		1.76 ± 0.09	1.76 ± 0.01	0.909
BMI (kg/m^2^, Mean ± SD)		25.4 ± 4.5	25.2 ± 3.2	0.866
Smoking (nonsmoker: smoker)∗		56 : 5	11 : 2	0.400
Alcohol consumption (Median [Q1-Q3]) ∗∗		3.0 [0.5-3.0]	2.0 [0-3.0]	0.590
Fazekas grade (Median [Q1-Q3]) ∗∗∗		0 [0-0]	0 [0-0]	0.177
Load of WMH (Median [Q1-Q3])		0 [0-1.0]	0 [0-3.5]	0.503
Number of infarctions		0	0	1.000
NIHSS (Median [Q1-Q3])		0 [0-0]	0 [0-0]	0.422
Previous conditions (n, %)	Cardiovascular	7, 11.5%	4, 30.8%	0.110
	Neuro-psychiatric	7, 11.5%	4, 30.8%	0.141
	Metabolic	14, 23%	1, 8%	0.256
	Autoimmune/Inflammatory	8, 13.1%	4, 30.8%	0.156

Summary of demographic, clinical, and risk factor variables stratified by MoCA scores (≥26 - cognitively normal; <26 - mild cognitive impairment). Continuous variables are presented as mean ± standard deviation (SD), or as median [1st quartile – 3rd quartile] if ordinal. Categorical variables are presented as absolute counts. Group comparisons were performed using unpaired t-tests for continuous variables and chi-square tests for categorical variables (sex and smoking status, marked with ∗). Alcohol consumption∗∗ was self-reported and categorized as follows: 0 = never, 1 = very rarely, 2 = rarely, 3 = occasionally, and 4 = every second day. Fazekas scores assessed the burden of deep white matter hyperintensities (DWMH) according to the following criteria: 0 = absence, 1 = punctate foci, 2 = beginning confluence of foci, 3 = large confluent areas. Load of DWMH was graded as: 0 = none, 1 = 1–5 lesions, 2 = 6–10 lesions, 3 = 11–25 lesions, 4 = >25 lesions. No infarctions were observed, and NIHSS scores (National Institutes of Health Stroke Scale) were close to zero across all participants. Pre-existing medical conditions were categorized into cardiovascular, neuro-psychiatric, metabolic, and autoimmune/inflammatory domains and are presented as counts and percentages. No statistically significant differences were found between groups across any demographic, clinical, or risk factor variables.

**Fig. 1:**
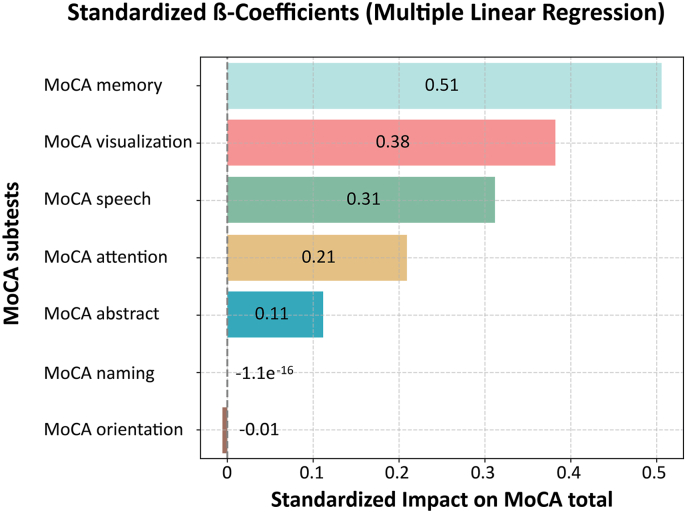
Standardized β-coefficients from multiple linear regression predicting MoCA total score This figure illustrates the standardized beta coefficients from a multiple linear regression model predicting the MoCA total score based on individual subtest performance. All variables were z-standardized prior to analysis to allow direct comparison of effect sizes. A vertical dashed line at zero represents the threshold between positive and negative influence.

Lower MoCA scores in the impaired group were primarily driven by deficits in visuospatial skills, memory, and language. A multiple linear regression using MoCA subtest scores revealed high explanatory power (R^2^ = 0.95), indicating that these three domains accounted for the majority of variance in total MoCA scores. Memory (β = 0.51), visuospatial skills (β = 0.38) and language (β = 0.31), emerged as the strongest predictors (Fig. 1).

### 3.2 QT1 and volumetric analyses between subjects with and without MCI

Automated segmentation revealed significantly lower absolute volumes in the left hippocampus (p = 0.019), left accumbens (p = 0.033), left thalamus (p = 0.028), and right thalamus (p = 0.046) in participants with MoCA <26 (Supplementary Table 1). After normalization for total intracranial volume (TIV), only the left hippocampus remained significantly reduced (p = 0.047), with trend-level reductions in the left accumbens (p = 0.074) and left amygdala (p = 0.085) (Supplementary Table 2). TIV did not differ between groups (p = 0.420), ruling out global size as a confounder. Detailed numerical values for regional volumetric and qT1 measurements are provided in Supplementary Table 1 and 2

In contrast, qT1 values did not differ significantly between groups in any region (all p > 0.050), suggesting preserved tissue properties despite localized volume loss (Supplementary Table 1). Again, no significant group differences were seen in vascular or systemic comorbidities, minimizing the risk of clinical confounding.

### 3.3 Relationships between qT1 metrics, MoCA scores and demographic factors

**Fig. 2:**
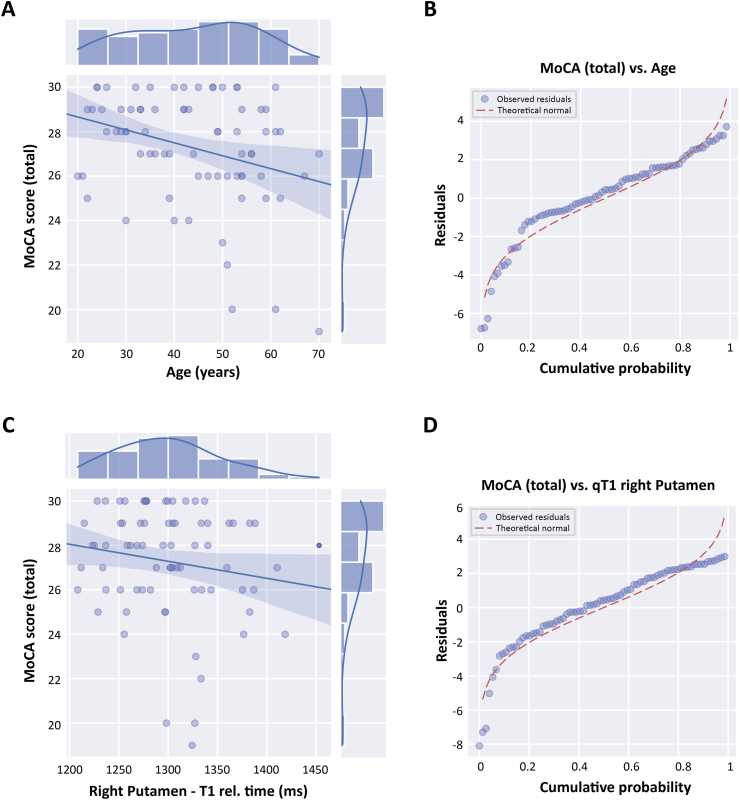
Relationship between age, right Putamen qT1 and total MoCA scores. (a, c) Scatterplots with marginal histograms and regression lines illustrate the relationships between total MoCA scores and (a) participant age and (c) quantitative T1 (qT1) relaxation times in the right putamen. MoCA scores decreased significantly with increasing age, and a trend toward lower MoCA scores with shorter right putamen qT1 times was observed. (b, d) Q-Q plots of the standardized residuals from the corresponding linear regression models show the alignment of residuals with the expected normal distribution. The residuals of the MoCA vs. Age model (b) show moderate deviation from normality, while the MoCA vs. qT1 in the right putamen model (d) exhibits slightly more pronounced deviations, particularly in the tails. These plots support the approximate adequacy of linear modeling, despite some non-normality in residual distribution.

A backward stepwise regression model identified age and qT1 in the right putamen as significant independent predictors of total MoCA score. While right putamen qT1 did not correlate significantly with MoCA in isolation (r = −0.16, p = 0.165, Fig. 2C–D), it gained predictive significance in the multivariate model adjusted for age. Age itself showed a significant negative correlation with MoCA (r = −0.31, p = 0.007, Fig. 2A–B), and also correlated negatively with right putamen qT1 (r = −0.38, p = 0.0009, Supplementary Fig. 1). However, variance inflation factors were low (both 1.17), indicating no concerning multicollinearity. Residuals from the model followed a normal distribution (Fig. 2B–D) supporting model adequacy. Finally, broader demographic and clinical variables — including sex, BMI, smoking status, alcohol consumption, cardiovascular, neuropsychiatric, metabolic, and autoimmune disease burden — were not significant predictors and were excluded during stepwise reduction.

**Fig. 3:**
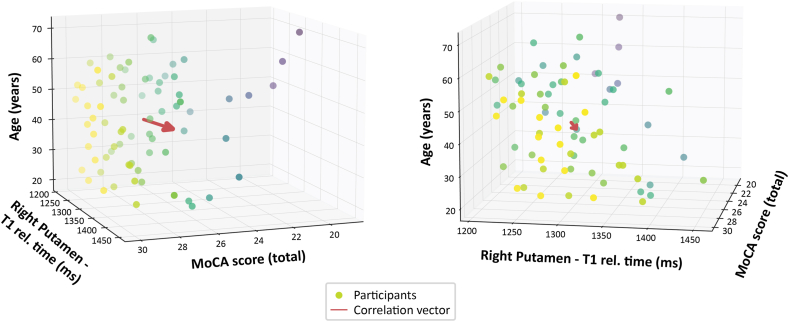
The 3D relationship between age, right putamen qT1, and MoCA Score. Three-dimensional scatterplots illustrating the relationship between age, right putamen qT1 relaxation time, and total MoCA score. Each point represents an individual participant. The red arrow indicates the overall direction of the correlation trend between the three variables. The color of the scatter points represents the total MoCA score, with brighter colors corresponding to higher scores and darker colors indicating lower scores.

To visualize these interactions, 3D scatterplots illustrate how lower MoCA scores are associated with both increasing age and decreasing qT1 in the right putamen (Fig. 3). The vector plot shows the joint influence of both predictors, while the alternate view highlights the qT1–age interaction. These data suggest that qT1, particularly in the right putamen, contributes explanatory value beyond age alone.

Additionally, age was significantly associated with lower cortical gray matter qT1 (r = −0.33, p = 0.005; Supplementary Fig. 2) and increased CSF volume (r = 0.45, p < 0.0001; Supplementary Fig. 3), consistent with age-related tissue remodeling. Variance inflation factors (VIF) for age, CSF volume, and cortical qT1 were all below 1.3 (VIF: age 1.14, CSF 1.28, qT1 GM 1.14), supporting the statistical independence of each factor. Together, these results confirm that age contributes uniquely to structural and subtle tissue changes relevant to cognition.

### 3.4 Relationships between volumetric metrics, MoCA scores and demographic factors

**Fig. 4:**
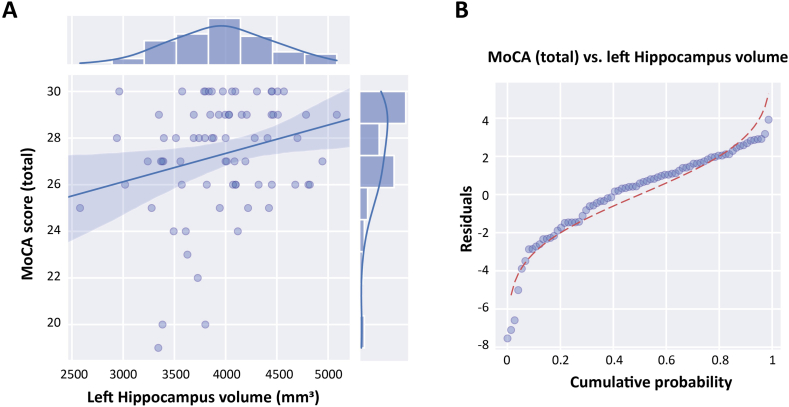
Relationship between volume of left hippocampus and total MoCA scores. (a) A scatterplot with marginal histograms and a fitted regression line illustrates the positive association between total MoCA score and left hippocampal volume (in mm^3^). Higher hippocampal volume is associated with better cognitive performance, as reflected by higher MoCA scores. (b) The Q-Q plot of residuals from the corresponding linear regression model shows the alignment between observed residuals and the theoretical normal distribution. While the residuals approximate normality, some deviations—especially in the lower tail—are visible. This supports the general adequacy of the linear model, though with minor distributional skew.

To further explore structural correlations of cognition, a backward stepwise regression revealed that left hippocampal volume was a significant predictor of MoCA scores (Fig. 4A). This relationship remained stable after adjustment for covariates. Residuals were normally distributed (Fig. 4B), validating the model.

These findings align with group comparisons showing that lower volumes in the left hippocampus, left accumbens, and thalami are associated with lower MoCA scores (Supplementary Table 1, Supplementary Fig. 3). Importantly, none of the demographic or clinical variables—sex, BMI, smoking, alcohol use, vascular/metabolic disease burden—were significantly related to MoCA scores. Fazekas scores and lesion load also did not differ between groups. These variables were excluded during stepwise model reduction, reinforcing the conclusion that structural brain measures, rather than systemic or demographic factors, underline cognitive variation in this cohort.

## 4 Discussion

This study investigated the utility of qT1 relaxometry alongside synthetic MRI volumetrics as imaging markers for cognitive performance, using MoCA scores as primary outcome. Our findings reveal that both the right putamen qT1 and the volume of the left hippocampus significantly predict MoCA scores, with age serving as an independent determinant. While the association between hippocampal volume, age, and cognitive performance is well established (Gauthier et al., 2006; Mufson et al., 2012; Stewart et al., 2019; Thomann et al., 2018), this study introduces the novel finding that right putaminal qT1 is independently associated with MoCA scores, even in the absence of volumetric loss.

Although right putamen qT1 did not correlate with MoCA scores in isolation, it remained a significant predictor in multivariate models controlling for age. The low VIFs support the statistical independence of these predictors. The association may reflect subtle tissue changes not visible on conventional morphometric analysis, offering a more nuanced measure of tissue integrity. Importantly, no significant group-level qT1 differences were observed between individuals classified as MCI and non-MCI, suggesting that qT1 captures continuous biological variation across the cognitive spectrum rather than threshold-based group effects.

Our cohort, consisting of 61 cognitively normal participants and 13 with MoCA-defined MCI (e.g. 18%), was well-characterized across demographic and clinical variables (Langa and Levine, 2014; Gauthier et al., 2006). None of these factors differed significantly between groups, minimizing confounding and reinforcing the relationship between brain structure, qT1, and cognition. The left hippocampus, accumbens, and thalami were significantly smaller in the MCI group—mirroring previous work by Tuokkola et al. (2019), who linked deep gray matter atrophy to declines in memory and visuospatial performance in MCI and AD. These regions play critical roles in cognitive control, memory, and executive function (Gauthier et al., 2006; Mufson et al., 2012). As hemispheric asymmetry was not hypothesized a priori, the observed lateralization should be considered exploratory and interpreted with caution, as it may reflect functional specialization or sample-related variability. Additionally, we observed age-related decreases in cortical gray matter qT1 and increases in CSF volume (Supplementary Fig. 2–3), aligning with prior literature on brain aging (Seiler et al., 2020; Fujita et al., 2023). These trends, which were statistically independent, further affirm age as a key factor in both structural and functional decline.

Despite no group-wise differences in qT1, right putamen qT1 emerged as a meaningful predictor of MoCA in multivariate models without any volumetric difference. This supports qT1 as a potential imaging biomarker associated processes such as demyelination, oxidative stress, or subtle iron accumulation (Steen et al., 1997; Stüber et al., 2014; Weiskopf et al., 2021; Kupeli et al., 2020; Uchida et al., 2020). Studies have shown that increased iron deposition and oxidative stress shorten qT1 times—particularly in aging and neurodegenerative contexts (Eminian et al., 2018; Seiler et al., 2020; Okubo et al., 2017; Kupeli et al., 2020; Uchida et al., 2020; Vymazal et al., 1996). Although qT1 lacks pathophysiological specificity, its integration with complementary modalities (e.g., T2 relaxometry, quantitative susceptibility mapping) could enhance diagnostic precision. Importantly, quantitative T1 mapping can be incorporated into standard MRI protocols using vendor-provided sequences, supporting its feasibility as a quantitative marker of tissue alterations in clinical settings. Indeed, qT1 is known to reflect non-iron-based processes - for example, prolonged qT1 relaxation times have been linked to myelin loss in multiple sclerosis, caused by neuroinflammation or microglial activation (Jonkman et al., 2015). This aligns with emerging theories that early alterations in brain tissue composition may precede overt neuronal loss and macroscopic atrophy, reinforcing the potential of qMRI in early disease detection. Recent neuropathological and ultra-high-resolution MRI studies further support this concept, demonstrating that degeneration of perforant pathway fibers and entorhinal–hippocampal circuitry occurs early along the Alzheimer's disease continuum and correlates with tau pathology even before overt cortical atrophy becomes apparent (Uchida et al., 2024, 2025a, 2025b).

Interpretation of qT1 changes remains complex. Apparent qT1 values may also be influenced by tissue loss and partial volume effects, including increased extracellular water or partial contamination by cerebrospinal fluid. Moreover, myelin-related effects are likely more pronounced in white matter than in gray matter due to the substantially higher myelin content in white matter tissue. Some studies report conflicting results regarding water content and qT1 values (Fatouros et al., 1991; Neeb et al., 2006; Gelman et al., 2001), possibly due to methodological differences or varying tissue properties across brain regions (Steen et al., 1997; Okubo et al., 2017). This emphasizes the need for region-specific analyses and harmonized protocols.

### 4.1 Limitations

Our findings should be interpreted considering several limitations. The small sample size, while sufficient for exploratory analyses, limits generalizability and should be interpreted with caution. Still, small cohorts remain valuable for identifying effect trends (Marek et al., 2022). Although participants were free of major neurological conditions, unmeasured factors – such as dopaminergic signaling or subclinical inflammation – may have affected cognition and qT1 values.

The cross-sectional design limits causal inferences and temporal resolution of brain-cognition dynamics. Longitudinal studies are needed to determine whether qT1 changes precede cognitive decline. While MoCA is sensitive, it lacks the specificity of comprehensive neuropsychological testing and does not replace established clinical diagnostic criteria for MCI (Petersen et al., 1999; Albert et al., 2011), constraining interpretation of domain-specific structure-function associations. Protocol- or scanner-related qT1 measurement variability also cannot be fully excluded.

Importantly, qT1 represents an apparent relaxation parameter influenced by multiple factors (including tissue microstructure) and cannot be attributed to a single biological substrate. Our imaging protocol did not include susceptibility-weighted imaging, high-resolution vessel imaging, or systematic assessment of cerebral microangiopathy. These omissions constrain evaluation of vascular contributions to cognitive impairment, a known factor in mixed dementia and MCI (Gauthier et al., 2006). Future studies should incorporate vascular imaging and risk profiling to clarify the significance of qT1 alterations in deep gray matter.

### 4.2 Outlook

This study offers evidence that qT1 relaxometry may complement volumetric MRI in assessing early cognitive decline. While hippocampal volume remains a strong indicator, qT1 may capture early tissue alterations before gross atrophy becomes apparent. As MRI techniques advance, integrating quantitative parameters into clinical assessments could enhance early diagnosis and monitoring of neurodegenerative changes. Longitudinal studies with larger, diverse populations and multimodal imaging are needed to confirm qT1's predictive utility.

## CRediT authorship contribution statement

**Lora Kovacheva:** Writing – review & editing, Writing – original draft, Visualization, Formal analysis, Conceptualization. **Jan R. Schüre:** Software, Methodology, Data curation, Conceptualization. **Svenja Klinsing:** Writing – review & editing, Formal analysis, Conceptualization. **Rafael Willems:** Writing – review & editing. **Mario Balo:** Writing – review & editing, Conceptualization. **Ralf Deichmann:** Writing – review & editing, Supervision, Formal analysis, Data curation, Conceptualization. **Elke Hattingen:** Writing – review & editing, Validation, Supervision, Resources, Project administration, Data curation, Conceptualization. **Christophe T. Arendt:** Writing – review & editing, Visualization, Validation, Supervision, Project administration, Methodology, Investigation, Funding acquisition, Formal analysis, Data curation, Conceptualization.

## Consent to participate

All participants provided written informed consent to participate in the study.

## Ethics approval

The study was approved by the Ethics Committee of the Faculty of Medicine at Goethe University Frankfurt, Germany (reference number: 20-838) and registered in the German Clinical Trials Register (Clinical trial number: DRKS00023880, https://drks.de/search/en, Date of Registration: 2021-01-07). It was conducted in accordance with the ethical principles outlined in the Declaration of Helsinki. All participants provided written informed consent for participation in the study. The scientific guarantor of this publication is Prof Dr Elke Hattingen.

## Consent for publication

All participants provided written informed consent for the publication of anonymized data.

## Code availability

Codes generated during the current study are available from the corresponding author on reasonable request.

## Funding

This study has received funding by a grant from the German Ministry of Education and Research via the German Center for Infection Research (DZIF) to Prof Dr Vehreschild and by the Goethe University Frankfurt via the Goethe Coronavirus Fund to Prof Dr Hattingen and Dr Arendt.

## Declaration of competing interest

The authors declare that they have no known competing financial interests or personal relationships that could have appeared to influence the work reported in this paper.

## Data Availability

Data will be made available on request.

## References

[bib1] Albert M.S., DeKosky S.T., Dickson D., Dubois B., Feldman H.H., Fox N.C., Gamst A., Holtzman D.M., Jagust W.J., Petersen R.C. (2011). The diagnosis of mild cognitive impairment due to Alzheimer's disease: recommendations from the national institute on aging-alzheimer’s association workgroups on diagnostic guidelines for Alzheimer's disease. Alzheimer's Dementia.

[bib2] Callaghan M.F., Freund P., Draganski B., Anderson E., Cappelletti M., Chowdhury R., Diedrichsen J., Fitzgerald T.H.B., Smittenaar P., Helms G. (2014). Widespread age-related differences in the human brain microstructure revealed by quantitative magnetic resonance imaging. Neurobiol. Aging.

[bib3] Dicks E., Tijms B.M., ten Kate M., Gouw A.A., Benedictus M.R., Teunissen C.E., Barkhof F., Scheltens P., van der Flier W.M. (2018). Gray matter network measures are associated with cognitive decline in mild cognitive impairment. Neurobiol. Aging.

[bib4] Eminian S., Hajdu S.D., Meuli R.A., Maeder P., Hagmann P. (2018). Rapid high resolution T1 mapping as a marker of brain development: normative ranges in key regions of interest. PLoS One.

[bib5] Fatouros P.P., Marmarou A., Kraft K.A., Inao S., Schwarz F.P. (1991). In vivo brain water determination by T1 measurements: effect of total water content, hydration fraction, and field strength. Magn. Reson. Med..

[bib6] Folstein M.F., Folstein S.E., McHugh P.R. (1975). “Mini-mental state.” A practical method for grading the cognitive state of patients for the clinician. J. Psychiatr. Res..

[bib7] Fujita S., Mori S., Onda K., Hanaoka S., Nomura Y., Nakao T., Yoshikawa T., Takao H., Hayashi N., Abe O. (2023). Characterization of brain volume changes in aging individuals with normal cognition using serial magnetic resonance imaging. JAMA Netw. Open.

[bib8] Gauthier S., Reisberg B., Zaudig M., Petersen R.C., Ritchie K., Broich K., Belleville S., Brodaty H., Bennett D., Chertkow H. (2006). Mild cognitive impairment. Lancet.

[bib9] Gelman N., Ewing J.R., Gorell J.M., Spickler E.M., Solomon E.G. (2001). Interregional variation of longitudinal relaxation rates in human brain at 3.0 T: relation to estimated iron and water contents. Magn. Reson. Med..

[bib10] Gracien R.M., van Wijnen A., Maiworm M., Petrov F., Merkel N., Paule E., Steinmetz H., Knake S., Rosenow F., Wagner M. (2019). Improved synthetic T1-weighted images for cerebral tissue segmentation in neurological diseases. Magn. Reson. Imaging.

[bib11] Jonkman L.E., Soriano A.L., Amor S., Barkhof F., van der Valk P., Vrenken H., Geurts J.J.G. (2015). Can MS lesion stages be distinguished with MRI? A postmortem MRI and histopathology study. J. Neurol..

[bib12] Kupeli A., Kocak M., Goktepeli M., Karavas E., Danisan G. (2020). Role of T1 mapping to evaluate brain aging in a healthy population. Clin. Imag..

[bib13] Langa K.M., Levine D.A. (2014). The diagnosis and management of mild cognitive impairment: a clinical review. JAMA.

[bib14] Marek S., Tervo-Clemmens B., Calabro F.J., Montez D.F., Kay B.P., Hatoum A.S., Donohue M.R., Foran W., Miller R.L., Hendrickson T.J. (2022). Reproducible brain-wide association studies require thousands of individuals. Nature.

[bib15] Mufson E.J., Binder L., Counts S.E., Dekosky S.T., Detoledo-Morrell L., Ginsberg S.D., Ikonomovic M.D., Perez S.E., Scheff S.W. (2012). Mild cognitive impairment: pathology and mechanisms. Acta Neuropathol..

[bib16] Nasreddine Z.S., Phillips N.A., Bédirian V., Charbonneau S., Whitehead V., Collin I., Cummings J.L., Chertkow H. (2005). The Montreal cognitive assessment, MoCA: a brief screening tool for mild cognitive impairment. J. Am. Geriatr. Soc..

[bib17] Neeb H., Zilles K., Shah N.J. (2006). Fully-automated detection of cerebral water content changes: study of age- and gender-related H2O patterns with quantitative MRI. Neuroimage.

[bib18] Nöth U., Hattingen E., Bähr O., Tichy J., Deichmann R. (2015). Improved visibility of brain tumors in synthetic MP-RAGE anatomies with pure T1 weighting. NMR Biomed..

[bib19] Okubo G., Okada T., Yamamoto A., Fushimi Y., Okada T., Murata K., Togashi K. (2017). Relationship between aging and T1 relaxation time in deep gray matter: a voxel-based analysis. J. Magn. Reson. Imag..

[bib20] Petersen R.C., Smith G.E., Waring S.C., Ivnik R.J., Tangalos E.G., Kokmen E. (1999). Mild cognitive impairment: clinical characterization and outcome. Arch. Neurol..

[bib21] Preibisch C., Deichmann R. (2009). T1 mapping using spoiled FLASH-EPI hybrid sequences and varying flip angles. Magn. Reson. Med..

[bib22] Preibisch C., Deichmann R. (2009). Influence of RF spoiling on the stability and accuracy of T1 mapping based on spoiled FLASH with varying flip angles. Magn. Reson. Med..

[bib23] Seiler A., Schöngrundner S., Stock B., Nöth U., Hattingen E., Steinmetz H., Klein J.C., Baudrexel S., Wagner M., Deichmann R. (2020). Cortical aging - new insights with multiparametric quantitative MRI. Aging.

[bib24] Small G.W., Kepe V., Ercoli L.M., Siddarth P., Bookheimer S.Y., Miller K.J., Lavretsky H., Burggren A.C., Cole G.M., Vinters H.V. (2006). PET of brain amyloid and tau in mild cognitive impairment. N. Engl. J. Med..

[bib25] Steen R.G., Ogg R.J., Reddick W.E., Kingsley P.B. (1997). Age-related changes in the pediatric brain: quantitative MR evidence of maturational changes during adolescence. Am. J. Neuroradiol..

[bib26] Stewart R.A.H., Held C., Krug-Gourley S., Waterworth D., Stebbins A., Chiswell K., Hagstrom E., Armstrong P.W., Wallentin L., White H. (2019). Cardiovascular and lifestyle risk factors and cognitive function in patients with stable coronary heart disease. J. Am. Heart Assoc..

[bib27] Stüber C., Morawski M., Schäfer A., Labadie C., Wähnert M., Leuze C., Streicher M., Barapatre N., Reimann K., Geyer S. (2014). Myelin and iron concentration in the human brain: a quantitative study of MRI contrast. Neuroimage.

[bib28] Thomann A.E., Goettel N., Monsch R.J., Berres M., Jahn T., Steiner L.A., Monsch A.U. (2018). The Montreal cognitive assessment: normative data from a german-speaking cohort and comparison with international normative samples. J. Alzheimers Dis..

[bib29] Tuokkola T., Karrasch M., Koikkalainen J., Parkkola R., Lötjönen J., Löyttyniemi E., Hurme S., Rinne J.O. (2019). Association between deep gray matter changes and neurocognitive function in mild cognitive impairment and alzheimer's disease: a tensor-based morphometric MRI study. Dement. Geriatr. Cogn. Disord.

[bib30] Uchida T., Togashi H., Kuroda Y., Yamashita A., Itoh N., Haga K., Sadahiro M., Kayama T. (2020). In vivo analysis of redox status in organs–from bench to bedside. Free Radic. Res..

[bib31] Uchida Y., Nishimaki K., Soldan A., Moghekar A., Albert M., Oishi K. (2024). Acceleration of brain atrophy and progression from normal cognition to mild cognitive impairment.

[bib32] Uchida Y., Ho S.G., Albert M., Nishimaki K., Soldan A., Pettigrew C., Moghekar A., Wang M., Miller M.I., Oishi K. (2025). Change points for dynamic biomarkers in the Alzheimer ’ s disease pathological cascade : a 30-year cohort study.

[bib33] Uchida Y., Hou Z., Gomez-isaza L., Troncoso J.C., Miller M.I., Mori S., Luongo M., Oishi K. (2025). Quantification of perforant path fibers for early detection of Alzheimer ’ s disease.

[bib34] Van De Mortel L.A., Thomas R.M., Van Wingen G.A. (2021). Grey matter loss at different stages of cognitive decline: a role for the thalamus in developing alzheimer's disease. J. Alzheimers Dis..

[bib35] Venkatesan R., Lin W., Haacke E.M. (1998). Accurate determination of spin-density and T1 in the presence of RF- field inhomogeneities and flip-angle miscalibration. Magn. Reson. Med..

[bib36] Volz S., Nöth U., Rotarska-Jagiela A., Deichmann R. (2010). A fast B1-mapping method for the correction and normalization of magnetization transfer ratio maps at 3 T. Neuroimage.

[bib37] Volz S., Nöth U., Jurcoane A., Ziemann U., Hattingen E., Deichmann R. (2012). Quantitative proton density mapping: correcting the receiver sensitivity bias via pseudo proton densities. Neuroimage.

[bib38] Vymazal J., Brooks R.A., Baumgarner C., Tran V., Katz D., Bulte J.W.M., Bauminger E.R., Di Chiro G. (1996). The relation between brain iron and NMR relaxation times: an in vitro study. Magn. Reson. Med..

[bib39] Weiskopf N., Edwards L.J., Helms G., Mohammadi S., Kirilina E. (2021). Quantitative magnetic resonance imaging of brain anatomy and in vivo histology. Nat. Rev. Phys..

