# Supplementary material for: 3D quantitative MRI for cognitive performance: T1 relaxation times of the right putamen and volumes of the left hippocampus as key biomarkers
Source: Neuroimage Rep. 2026 May 7;6(2):100351. doi: 10.1016/j.ynirp.2026.100351 (PMC13185900; doi:10.1016/j.ynirp.2026.100351)
Supplement: Multimedia component 1 [file mmc1.docx]

**SuPPLEMENTARY DATA – 3D Quantitative MRI for Cognitive Performance:**

**T1 Relaxation Times of the Right Putamen**

**and Volumes of the Left Hippocampus as Key Biomarkers**

Lora Kovacheva*, Jan R Schüre, Svenja Klinsing, Rafael Willems, Mario Balo, Ralf Deichmann, Elke Hattingen, Christophe T Arendt**

*first and corresponding author

** last author

Lora Kovacheva, corresponding author: [lorask@gmail.com](mailto:lorask@gmail.com), ORCID 0000-0001-6999-1533

Jan R Schüre ORCID: 0000-0002-1472-9471

Svenja Klinsing ORCID: 0000-0001-5051-6839

Rafael Willems ORCID: 0009-0007-4976-6890

Mario Balo: 0009-0006-2212-4591

Ralf Deichmann ORCID: 0000-0002-4110-1225

Elke Hattingen ORCID: 0000-0002-8392-9004

Christophe T Arendt ORCID: 0000-0003-3300-3729

Institutional affiliation for all authors: Institut für Neuroradiologie, Universitätsklinikum Frankfurt, Frankfurt am Main, Germany

## **Key words**

Cognitive Dysfunction, qT1, MRI, Dementia, Putamen.

## **Supplementary Data**

##

Supplementary Table 1 - Group comparison of cognitive, raw volumetric, and qT1 parameters between participants with and without cognitive impairment

*This table presents the mean values of MoCA subtests, brain regional volumes, and qT1 relaxation times for two groups of participants: those with normal cognition (MoCA ≥ 26) and those with mild cognitive impairment (MoCA < 26). The groups were compared using multiple unpaired t-tests (Two-stage step-up, Benjamini, Krieger, and Yekutieli, FDR 10%). Statistically significant group differences (p < 0.050) were found in several MoCA subdomains, including visualization, attention, speech, and memory, with lower scores in the impaired group. Significant volumetric reductions were observed in the left hippocampus, left accumbens, left thalamus, and right thalamus in the MoCA < 26 group. No statistically significant differences in qT1 values were observed between groups.* *The rows with a statistically significant p-value were highlighted with a light gray background for emphasis. Legend: MoCA - Montreal Cognitive Assessment, CSF – cerebrospinal fluid, LGM – left grey matter, LWM – left white matter, RGM – right grey matter, RWM – right white matter, LTha – left thalamus, LCaud – left nucleus caudatus, LPut – left putamen, LPal – left globus pallidus, BSt4V/Brstem4Vent – brainstem with 4. ventricle, LHip – left hippocampus, LAmy – left amygdala, LAcc – left nucleus accumbens, RTha – right thalamus, RCaud – right nucleus caudatus, RPut – right putamen, RPal – right globus pallidus, RHip – right hippocampus, RAmy – right amygdala, RAcc – right nucleus accumbens, T1 – qT1 relaxation time.*

| Parameter | MoCA≥26 (mean ± SD) | MoCA <26 (mean ± SD) | p-value (t-test) |
| --- | --- | --- | --- |
| MoCA-visualization | 4.656 ± 0.629 | 3.538 ± 1.450 | <0.0001 |
| MoCA-naming | 3.0 ± 0 | 3 ± 0 | 1 |
| MoCA-attention | 5.787 ± 0.5 | 5.308 ± 0.8 | 0.005 |
| MoCA-speech | 2.738 ± 0.5 | 1.615 ± 0.96 | <0.0001 |
| MoCA-abstract | 1.902 ± 0.4 | 1.692 ± 0.5 | 0.073 |
| MoCA-memory | 4.115 ± 1.1 | 2.308 ± 1.6 | <0.0001 |
| MoCA-orientation | 5.984 ± 0.1 | 6 ± 0 | 0.648 |
| CSF (mm^3^) | 183753.6 ± 40260.5 | 200973 ± 62835.6 | 0.258 |
| LGM (mm^3^) | 217439.1 ± 18690.2 | 2078345 ± 17142.6 | 0.154 |
| LWM (mm^3^) | 196074.0 ± 17609.1 | 186352 ± 18459.3 | 0.162 |
| RGM (mm^3^) | 217520.5 ± 18879.4 | 206879 ± 16663.7 | 0.101 |
| RWM (mm^3^) | 195768.3 ± 17761.4 | 186119 ± 18703.7 | 0.172 |
| LTha (mm^3^) | 8073 ± 777.4 | 7481 ± 646.7 | 0.028 |
| LCaud (mm^3^) | 3720 ± 501.6 | 3516 ± 540.1 | 0.133 |
| LPut (mm^3^) | 5228 ± 596.4 | 5132 ± 690.5 | 0.599 |
| LPal (mm^3^) | 1778 ± 239.5 | 1677 ± 196.8 | 0.170 |
| BSt4V (mm^3^) | 22971 ± 2765.7 | 22506 ± 2888.4 | 0.574 |
| LHip (mm^3^) | 4015 ± 511.7 | 3658 ± 526.9 | 0.019 |
| LAmy (mm^3^) | 1259 ± 191.5 | 1362 ± 230.4 | 0.174 |
| LAcc (mm^3^) | 594.1 ± 119.2 | 526.5 ± 113.3 | 0.033 |
| RTha (mm^3^) | 7883 ± 756.4 | 7404 ± 706.1 | 0.046 |
| RCaud (mm^3^) | 3867 ± 508 | 3605 ± 474.1 | 0.061 |
| RPut (mm^3^) | 5047 ± 585.6 | 4893 ± 648.2 | 0.408 |
| RPal (mm^3^) | 1858 ± 232.5 | 1775 ± 221.7 | 0.276 |
| RHip (mm^3^) | 4091 ± 497.6 | 3862 ± 524.5 | 0.128 |
| Ramy (mm^3^) | 1236 ± 188.3 | 1222 ± 208.9 | 0.856 |
| Racc (mm^3^) | 494.8 ± 99.4 | 455.4 ± 90.7 | 0.240 |
| T1_CSF (ms) | 3295 ± 124.8 | 3313 ± 135.6 | 0.724 |
| T1_LGM (ms) | 1521 ± 46.9 | 1535 ± 58.2 | 0.350 |
| T1_LWM (ms) | 920.5 ± 24.7 | 932.8 ± 29.3 | 0.185 |
| T1_RGM (ms) | 1520 ± 47.1 | 1535 ± 59.1 | 0.320 |
| T1_RWM (ms) | 919.3 ± 24.5 | 930.5 ± 28.8 | 0.204 |
| T1_TotalWM (ms) | 919.9 ± 24.6 | 931.7 ± 29 | 0.184 |
| T1_LTha (ms) | 1268 ± 58.2 | 1306 ± 69.5 | 0.079 |
| T1_LCaud (ms) | 1404 ± 68.6 | 1424 ± 79 | 0.349 |
| T1_LPut (ms) | 1311 ± 60.4 | 1340 ± 74.6 | 0.083 |
| T1_LPal (ms) | 1056 ± 47.5 | 1079 ± 53.3 | 0.146 |
| T1_Brstem4Vent (ms) | 1359 ± 86.9 | 1389 ± 92.5 | 0.366 |
| T1_LHip (ms) | 1589 ± 79.7 | 1595 ± 86 | 0.777 |
| T1_Lamy (ms) | 1632 ± 83.6 | 1664 ± 95.4 | 0.212 |
| T1_LAcc (ms) | 1655 ± 92.1 | 1649 ± 99.6 | 0.836 |
| T1_RTha (ms) | 1298 ± 61.5 | 1320 ± 73.1 | 0.245 |
| T1_RCaud (ms) | 1388 ± 70 | 1386 ± 76.3 | 0.950 |
| T1_RPut (ms) | 1297 ± 59.6 | 1317 ± 71.8 | 0.206 |
| T1_RPal (ms) | 1036 ± 44.7 | 1039 ± 49.1 | 0.783 |
| T1_RHip (ms) | 1566 ± 76.3 | 1572 ± 83.7 | 0.779 |
| T1_RAmy (ms) | 1641 ± 86.8 | 1630 ± 91.4 | 0.719 |
| T1_RAcc (ms) | 1634 ± 88.9 | 1650 ± 96.1 | 0.652 |

Supplementary Table 2 - Comparison of TIV-normalized subcortical brain volumes between cognitively normal and impaired individuals

*This table displays the comparison of total intracranial volume (TIV) and TIV-normalized volumes of bilateral subcortical brain regions between participants with normal cognitive function (MoCA ≥ 26) and those with mild cognitive impairment (MoCA < 26). Volumes were normalized by each subject's TIV to account for individual differences in head size. Group means and p-values from multiple unpaired t-tests (Two-stage step-up, Benjamini, Krieger, and Yekutieli, FDR 10%) are reported. A significant reduction was observed in the left hippocampus (p = 0.047) in the MoCA < 26 group. Trends toward lower normalized volumes in the left accumbens and left amygdala were also observed, though not reaching significance. No significant differences were found in TIV or other subcortical structures.* *The row with a statistically significant p-value is highlighted with a light gray background for emphasis. Legend: LTha – left thalamus, LCaud – left nucleus caudatus, LPut – left putamen, LPal – left globus pallidus, BSt4V– brainstem with 4. ventricle, LHip – left hippocampus, LAmy – left amygdala, LAcc – left nucleus accumbens, RTha – right thalamus, RCaud – right nucleus caudatus, RPut – right putamen, RPal – right globus pallidus, RHip – right hippocampus, RAmy – right amygdala, RAcc – right nucleus accumbens.*

| Parameter | MoCA ≥26 (mean ± SD) | MoCA <26 (mean ± SD) | p-value (t-test) |
| --- | --- | --- | --- |
| TIV | 1010555 ± 99139.206 | 988158 ± 89332.326 | 0.417 |
| LTha (norm) | 0.008001 ± 0.000770 | 0.007605 ± 0.000733 | 0.068 |
| LCaud (norm) | 0.003699 ± 0.000415 | 0.003572 ± 0.000409 | 0.368 |
| LPut (norm) | 0.005185 ± 0.000560 | 0.005222 ± 0.000568 | 0.815 |
| LPal (norm) | 0.001766 ± 0.000251 | 0.001706 ± 0.000246 | 0.401 |
| BSt4V (norm) | 0.022738 ± 0.001612 | 0.022831 ± 0.001514 | 0.868 |
| LHip (norm) | 0.003979 ± 0.000518 | 0.003720 ± 0.000518 | 0.047 |
| LAmy (norm) | 0.001252 ± 0.000156 | 0.001383 ± 0.000164 | 0.085 |
| LAcc (norm) | 0.000588 ± 0.000090 | 0.000537 ± 0.000096 | 0.074 |
| RTha (norm) | 0.00782 ± 0.000748 | 0.007522 ± 0.000727 | 0.144 |
| RCaud (norm) | 0.003843 ± 0.000372 | 0.003662 ± 0.000363 | 0.198 |
| RPut (norm) | 0.005004 ± 0.000674 | 0.004980 ± 0.000683 | 0.888 |
| RPal (norm) | 0.001843 ± 0.000244 | 0.001801 ± 0.000239 | 0.537 |
| RHip (norm) | 0.004056 ± 0.000533 | 0.003932 ± 0.000548 | 0.348 |
| RAmy (norm) | 0.001225 ± 0.000287 | 0.001251 ± 0.000293 | 0.725 |
| RAcc (norm) | 0.000489 ± 0.000060 | 0.000462 ± 0.000062 | 0.363 |


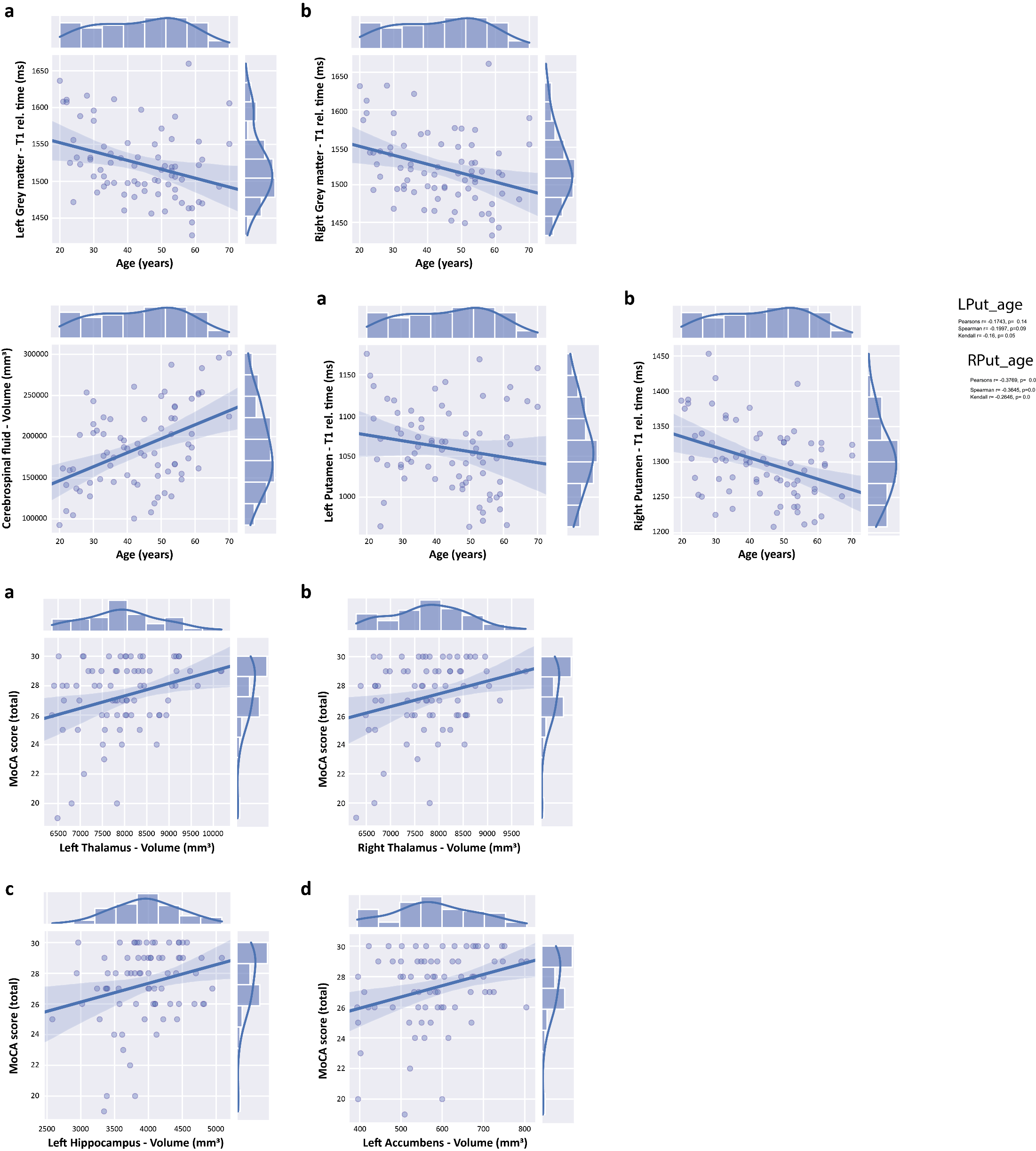


Supplementary Figure 1

*Scatterplot with marginal histograms describing the correlation between left (a) and right (b) putamen qT1 relaxation times, against subject’s age. Statistics: (a) Pearsons’ r = -0.17, p= 0.14; (b) Pearsons’ r = -0.38, p = 0.0009.*


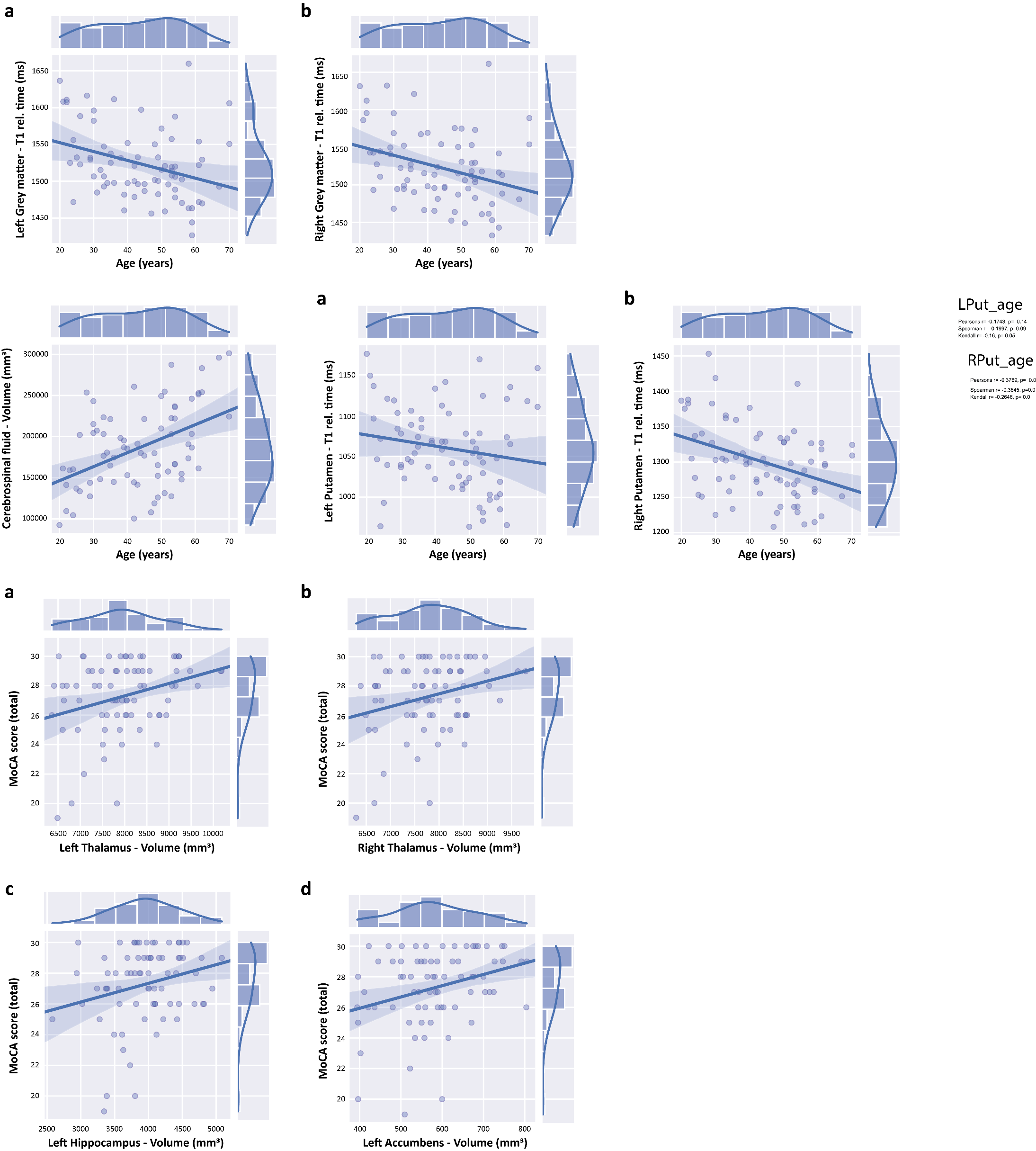


Supplementary Figure 2

*Scatterplot with marginal histograms describing the correlation between qT1 relaxation time of the left grey matter (a) and right grey matter (b) against age. Abbreviation: rel. – relaxation. Statistics: (a) Pearsons’ r = -0.3251, p = 0.005; (b) Pearsons’ r = -0.3234, p = 0.005*


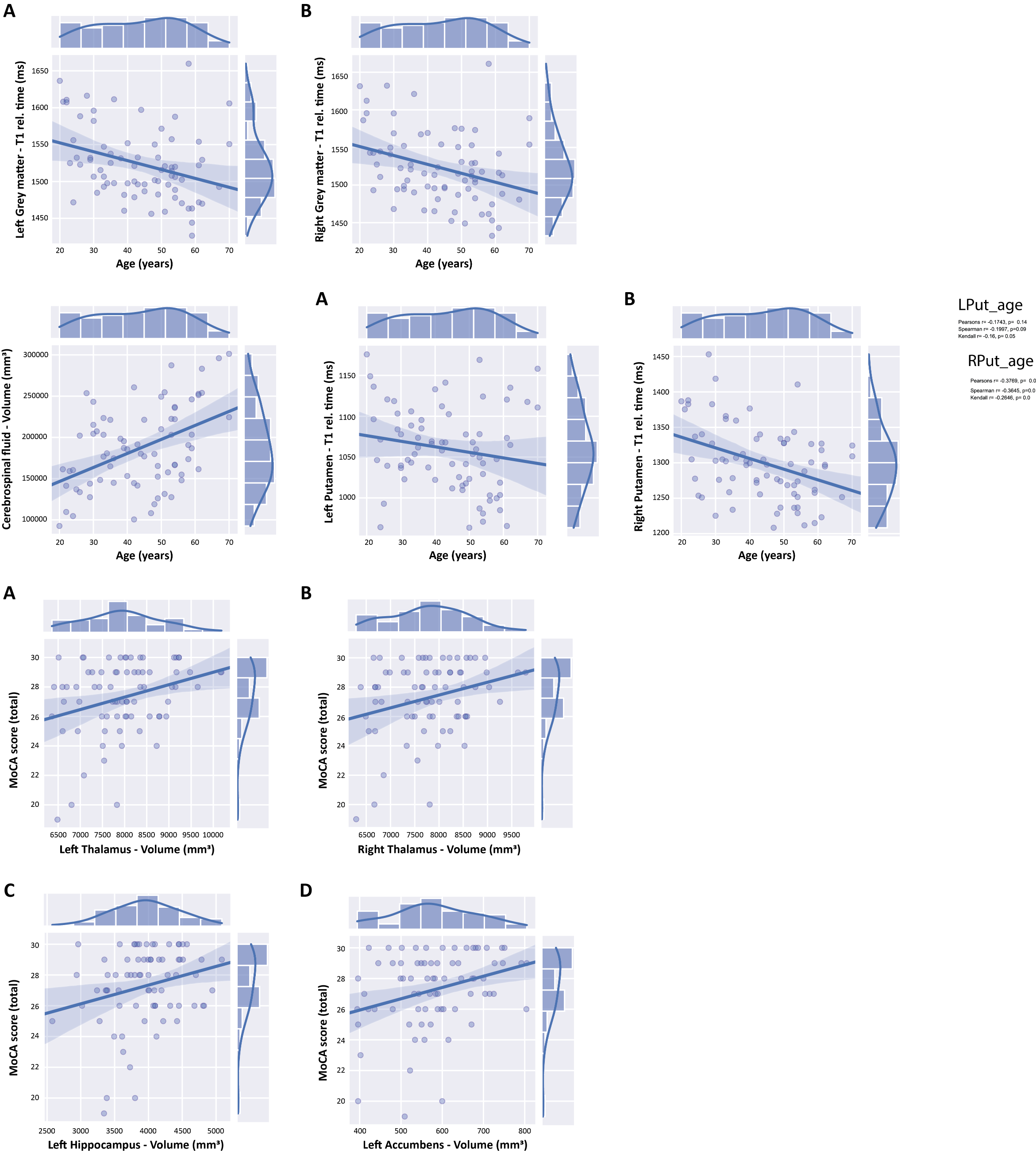


Supplementary Figure 3

*Scatterplot with marginal histograms describing the correlation between volume of cerebrospinal fluid and age. Statistics: Pearsons’ r = 0.4531, p < 0.0001*


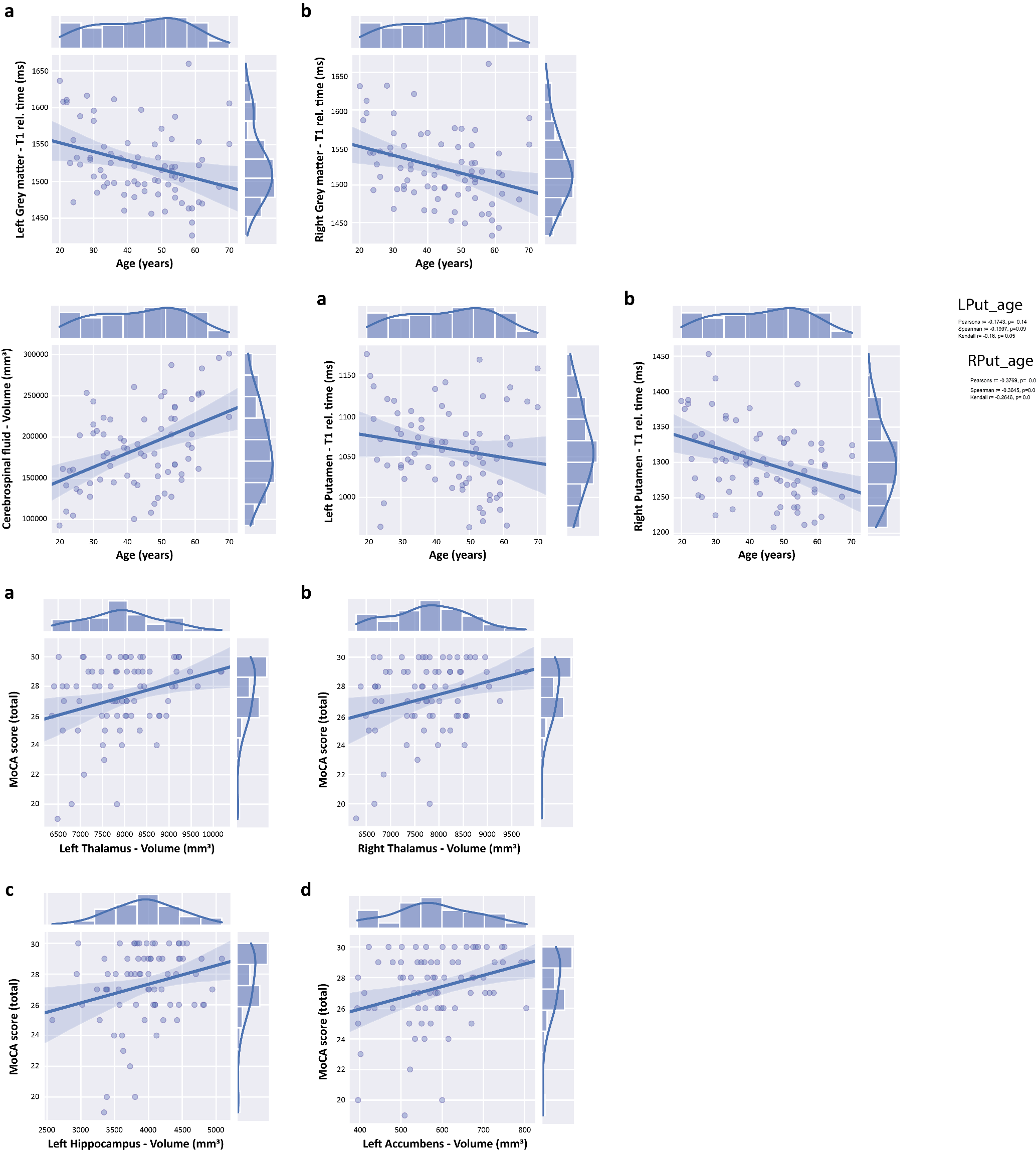


Supplementary Figure 4

*Scatterplot with marginal histograms describing the correlation between volumes of the left thalamus (A), right thalamus (B), left hippocampus (C) and left accumbens (D) against total MoCA score. Statistics: (A) Pearsons’ r = 0.3023, p= 0.010; (B) Pearsons’ r = 0.2779, p = 0.02; (C) Pearsons’ r = 0.247, p= 0.03; (D) Pearsons’ r = 0.3106, p= 0.010*

## **Declarations**

**Funding**

This study has received funding by a grant from the German Ministry of Education and Research via the German Center for Infection Research (DZIF) to Prof Dr Vehreschild and by the Goethe University Frankfurt via the Goethe Coronavirus Fund to Prof Dr Hattingen and Dr Arendt.

**Conflicts of interest / Competing interests**

The authors declare that they have no competing interests.

**Ethics approval**

The study was approved by the Ethics Committee of the Faculty of Medicine at Goethe University Frankfurt, Germany (reference number: 20-838) and registered in the German Clinical Trials Register (Clinical trial number: DRKS00023880, <https://drks.de/search/en>, Date of Registration: 2021-01-07). It was conducted in accordance with the ethical principles outlined in the Declaration of Helsinki. All participants provided written informed consent for participation in the study. The scientific guarantor of this publication is Prof Dr Elke Hattingen.

**Consent to participate**

All participants provided written informed consent to participate in the study.

**Consent for publication**

All participants provided written informed consent for the publication of anonymized data.

**Availability of data and materials**

The datasets generated and analyzed during the current study are available from the corresponding author on reasonable request.

**Code availability**

Codes generated during the current study are available from the corresponding author on reasonable request.

**Author contributions**

All authors read and approved of the final manuscript. EH and CA contributed to this work by handling conceptualization, funding acquisition, methodology, project administration, supervision, validation, and editing the original draft. LK and CA contributed to this work by handling conceptualization, data curation, formal analysis, investigation, validation, visualization, and writing the original draft. JR and RD contributed to this work by handling conceptualization, data curation, methodology, supervision, validation, and editing the original draft. SK, MB and RW contributed to this work by handling data curation, project administration, validation, and editing the original draft.
